# MRI in Oncology

**Published:** 1988-05

**Authors:** P. Goddard


### MRI in Oncology

Magnetic Resonance Imaging is potentially useful in Oncology in a large number of ways. The possibilities include screening, diagnosis, staging, radiotherapy and surgery planning and the demonstration of recurrence and metastases.

The lack of harmful effects would make MRI highly suitable for screening if the technique was more widely available and less expensive. The STIR sequence, described in one of the following articles, is a method of demonstrating lesions with considerable contrast to the surrounding normal structures and as such may be applicable to screening for neoplasia. Because of the present cost of the technique the application of MRI in screening has not been explored.

The use of MRI in the primary diagnosis of suspected malignancy is already well accepted in the CNS. This is discussed, with many examples, in the article by Bradshaw and Lewis. The value of MRI in the primary diagnosis of malignancy in the body has not been clearly established.

Presently the main role of MRI in Oncology outside the CNS appears to be in the demonstration of extent of known disease for staging and treatment planning and the detection of recurrence and metastases. It has been shown that MRI is superior to CT when studying primary bone tumours and this can be very important now that prosthetic replacement is being offered in treatment rather than amputation. The detection of recurrence and metastases is described in the following papers on the pelvis and the spine. Determination of the extent of malignant disease is also discussed with regard to the chest.

Radiotherapy planning applications are already becoming clear. The facility of scanning in any desired plane, with equal resolution, is clearly an advantage over CT. This is demonstrated in the case of rhabdomyosarcoma of the orbit discussed in this issue in the paper by Berger et al. The high contrast between lesions and surrounding tissues enables decisions over radiotherapy fields to be made with greater precision.

